# Comparative analysis of the tear protein profile in mycotic keratitis patients

**Published:** 2008-03-12

**Authors:** Sivagnanam Ananthi, Thangavel Chitra, Ramachandran Bini, Namperumalsamy Venkatesh Prajna, Prajna Lalitha, Kuppamuthu Dharmalingam

**Affiliations:** 1 Dr.G.Venkataswamy Eye Research Institute, Aravind Medical Research Foundation, Aravind Eye Care System, Madurai, India; 2 Cornea Clinic, Aravind Eye Hospital, Aravind Eye Care System, Madurai, India; 3 Department of Microbiology, Aravind Eye Hospital, Aravind Eye Care System, Madurai, India; 4 School of Biotechnology, Madurai Kamaraj University, Madurai, India

## Abstract

**Purpose:**

Mycotic keratitis is a major cause of corneal blindness in India. A proper understanding of the pathogenesis may help in refining the existing treatment. The purpose of this study is to examine the total tear protein profile of fungal keratitis patients, which may have a bearing on pathogenesis and disease progression.

**Methods:**

Tear samples were collected from culture positive fungal keratitis patients. Tears from the uninfected fellow eye and from other healthy individuals served as controls. Two-dimensional electrophoresis (2DE) was used for the separation of fractionated tear proteins, and selected protein spots, which showed differential expressions, were identified using matrix-assisted laser desorption/ionization-time of flight (MALDI-TOF) mass spectrometry. Wherever needed, tag sequencing of peptide fragments using post source decay (PSD) was done to confirm the identification.

**Results:**

The glutaredoxin-related protein was expressed only in the tears of fungal keratitis patients. Six other normal tear proteins were present in both samples but with varied expression levels. Prolactin inducible protein and serum albumin precursor were upregulated in the infected samples. Cystatin S precursor, cystatin SN precursor, cystatin, and human tear lipocalin were downregulated in the infected samples.

**Conclusions:**

Tears can be used as a clinical source to study the proteomic responses in patients with fungal keratitis. The glutaredoxin-related protein is known to be produced by *Aspergillus* during oxidative stress conditions, and the presence of this protein in the tears of patients with mycotic keratitis indicates that this pathogen undergoes stress-related gene expression during infection.

## Introduction

In developing countries, fungi have replaced bacteria as the most common cause of suppurative keratitis [1,2]. Very recently, fungi have been implicated as the causative organism in a series of contact lens-related infectious keratitis in the United States as well [3-5]. The visual outcome following mycotic keratitis is generally poorer than bacterial keratitis, probably due to the lack of effective antifungal therapy [6]. There is yet no effective validated antifungal drug sensitivity assessment [7]. While newer and newer antibacterial drugs continue to be discovered, natamycin, which was discovered in the 1960s, still remains the drug of choice for the treatment of fungal keratitis [8]. Therapeutic keratoplasty performed for mycotic keratitis is known to have a poorer prognosis than similar surgeries done for bacterial keratitis [9].

Ocular fungal infections or ophthalmic mycoses are being increasingly recognized as the cause of morbidity and blindness, and certain types of ophthalmic mycoses may even be life threatening [10,11]. Fungi are opportunistic in the eye since they rarely infect healthy, intact ocular tissues. Even the trivial trauma of a dust particle falling on the cornea may disrupt the integrity of the corneal epithelium, predisposing it to mycotic keratitis [12]. The key agent factors involved in the pathogenesis of mycotic keratitis include adherence, invasiveness, morphogenesis, and toxigenicity. Fungi that causes keratitis, in particular *Fusarium* sp., sometimes invade the anterior chamber and form a lens-iris-fungus mass at the pupillary area, thereby interfering with the normal drainage of the aqueous humor and leading to a rise in intraocular pressure [13,14]. The morphogenetic and phenotypic switching permit fungi to adapt in different microenvironments and to survive in the infected host [15]. The presence of intrahyphal hyphae or hypha-in-hyphae and thickened fungal cell walls may reflect such morphogenesis occurring in fungi that is invading corneal tissue; these morphological alterations may constitute a barrier against antifungal drugs or host defenses [16,17] or may function as virulence factors of fungi in corneas where the defense mechanism have been compromised by the application of corticosteroids [18]. Mycotoxin production may also play an important role in the pathogenesis [12].

It is imperative that the functional and molecular aspects of the disease be well understood to develop an effective treatment plan. Toward this, we have reported earlier the usefulness of tear samples in understanding the cytokine response in fungal keratitis [19]. As a sequel to this, we used a proteomic approach to compare the tear protein profile of patients with that of controls.

Proteome analysis has emerged as a powerful approach to complement transcript analysis to examine the mechanisms of complex multivariate diseases at the functional molecular level. The ability to analyze posttranslational modifications makes proteome analysis a necessary approach to disease-specific changes.

The proteomic analysis of tears already showed promising results in eye research [20-22]. The tear proteins play an important role in maintaining the ocular surface, and changes in tear protein components may reflect the changes in the health of the ocular surface [23,24]. Proteomic analysis can provide more insights about protein expression patterns, which are associated with various pathological conditions, and the identification of those tear proteins and their posttranslational modifications have the potential to reveal the mechanism of eye disease [23,25,26].

Recent advances in mass spectrometric technology, particularly in matrix-assisted laser desorption/ionization-time of flight (MALDI-TOF) and liquid chromatography tandem mass spectrometry (LC MS/MS) techniques complement and expand the scope of two-dimensional gel electrophoresis in proteome analysis [27]. In this paper, we used these techniques to examine the alteration in the tear proteome in fungal keratitis and this is the first report as far as we are aware of.

## Methods

### Tear sample collection

IRB approval from the Aravind Medical Research Foundation (Madurai, India) was obtained for this study. The Declaration of Helsinki was adhered to when enrolling subjects. After informed consent, reflex tear samples (100–150 μl) were collected from the infected eye of culture positive (*Aspergillus* or *Fusarium*) fungal keratitis patients. Tears from the fellow eye, which was not infected, and from other healthy individuals served as controls (60–80 μl).

Tear samples were collected using 10 µl-capillary tubes without touching the eye globe or the lid. The samples were centrifuged at 7800x g for 10 min at 4 °C to remove cellular debris and stored in liquid nitrogen until analysis. Samples were collected from patients before treatment. Protein was estimated using Bradford’s method [28].

### Two-dimensional electrophoresis (2DE) analysis of tear proteins

#### Sample preparation

Tear samples from 16 *Fusarium* keratitis patients and 16 *Aspergillus* keratitis patients were pooled (300 µg of protein per patient). Tear samples from 24 healthy individuals were pooled and used as control samples (200 µg of protein per healthy individual). Tear samples from the fellow eye were insufficient, hence we could not use these as control subjects. Pooled tear samples were concentrated using the deoxycholate precipitation protocol [29] with minor modifications. Briefly, 500 μl of sample was treated with 5 μl of 2% deoxycholate (prepared in water), vortexed well, and incubated at room temperature for 10 min. To the mixture, 50 μl of chilled trichloroacetic acid was added, vortexed well, and incubated in ice for 15 min followed by centrifugation at 7800x g for 10 min at 4 °C. The supernatant was removed carefully and 200 μl of chilled acetone was added to the pellet, vortexed, and incubated in ice for 10 min followed by centrifugation at 7800x g for 10 min at 4 °C. The supernatant was removed and the pellet was dried at room temperature for 20 min. The dried pellet was resuspended in 50 μl of TE buffer (25 mM Tris, pH 7.4; 50 mM EDTA). Protein concentration was usually about 8 μg/μl.

#### Isoelectric focusing

The protein concentration used for preparative gels to be stained with colloidal coomassie blue G-250 was 270 μg, and for the MALDI compatible silver staining method, 50 μg of protein was used. For rehydration, protein was diluted to 350 μl in a buffer containing 7 M Urea, 2 M thiourea, 4% CHAPS, 0.5% ampholytes, 50 mM DTT, and 0.004% of bromophenol blue. Samples were loaded using the passive rehydration method for a minimum of 16 h. Rehydrated strips were rinsed with water and then focused at a temperature of 20 °C using the IPGphor IEF apparatus (GE Healthcare, Hong Kong, China) using the following voltage settings: 500 V for 1 h (step and hold), 1000 V for 1 h (gradient), 8000 V for 3 h (gradient), and 8000 V for 8 h (step and hold). The focused strips were stored at −70 °C until the two-dimensional analysis or were used immediately.

#### SDS–PAGE

The strips were brought to room temperature and equilibrated for 15 min each to reduce and alkylate the proteins. The equilibration buffer contained 6 M Urea, 2 M Thiourea, 50 mM Tris, 34.5% glycerol, 2% SDS, and 0.005% bromophenol blue with 2% DTT for the first step, and 2.5% iodoacetamide replaced DTT for the second step. Strips were then placed on top of a 12.5% polyacrylamide gel and sealed with 0.5% agarose dissolved in electrophoresis buffer. The DALT six apparatus (GE Healthcare, Hong Kong, China) was used for 2DE a constant temperature of 22 °C (20 W for 30 min and 60 W for 5 h). Experimental and control samples were examined in parallel to minimize experimental variations.

#### Staining

Gels were stained using colloidal coomassie blue G-250 [30] and the MALDI compatible silver staining method [31] (with minor modifications). For colloidal coomassie staining, the gels were fixed in 40% methanol and 10% acetic acid in water for 1 h. Gels were washed three times in water for 10 min each and then stained overnight in a staining solution containing 10% phosphoric acid, 10% ammonium sulfate, 0.12% coomassie blue G-250, and 20% methanol. Gels were then destained in water.

For MALDI compatible silver staining, gels were fixed in 40% ethanol and 10% acetic acid in water for 1 h. The gels were then rinsed in 30% ethanol for 20 min. Gels were given three water washes for 10 min each and then sensitized using 0.02% sodium thiosulfate for 1 min. After water wash for 10 s, gels were developed in 2% sodium carbonate and 0.04% formalin followed by another water wash for 20 s. Development was stopped with 5% acetic acid solution and then the stained gels were washed three times in water for 10 min each.

#### In-gel tryptic digestion

In-gel digestion was done essentially as described earlier [32]. In brief, the gel pieces were first washed two times in water for 10 min each. The pieces were destained using 25 mM ammonium bicarbonate made in 50% acetonitrile. The pieces were destained in 3 changes of the solution (15 min each change). After dehydration in 100% acetonitrile for 15 min, the gel pieces were dried under vacuum for 30 min. Dried gel pieces were rehydrated for 30 min on ice with 400 ng of trypsin dissolved in 5 µl of 100 mM ammonium bicarbonate in 10% acetonitrile. Excess trypsin was then removed, and the gel pieces were covered with 20 µl of 40 mM ammonium bicarbonate in 10% acetonitrile and incubated at 37 °C for 16 h. The tubes were then briefly centrifuged for 5 s and the supernatant saved. Peptides were then eluted from gel pieces using 25 µl of 0.1% trifluoroacetic acid (TFA) made in 60% acetonitrile by sonication for 3 min at a frequency of 2200 MHz in the Soltech ultrasonic cleaner (Soltec,Milano,Italy). The tubes were then centrifuged for 5 s and incubated for 10 min. The supernatant obtained after centrifugation was added to the first supernatant. Acetonitrile (20 μl of 100%) was added to gel pieces for dehydration and vortexed. The tubes were then centrifuged for 5 s and incubated for 10 min. The supernatant was combined with the first two supernatants, and these extracted peptides were then dried under vacuum. Dried peptides were dissolved in 5 µl of 0.1% TFA in 5% acetonitrile. Samples were desalted using Eppendorf perfect pure C18 tips and eluted in 5 µl of 0.1% TFA in 50% acetonitrile according to the manufacturer’s instructions.

### MALDI-TOF spectrometry of tryptic digests

A MALDI-TOF analysis of extracted tryptic peptides was done as described earlier [32]. The matrix used in this study was the α–cyano-4-hydroxy cinnamic acid (CHCA) matrix, which was dissolved in 70% acetonitrile and 0.03% TFA at a concentration of 2 mg/ml. In some experiments, 50 mM ammonium monobasic phosphate was used as an additive. Peptide samples were applied on a stainless steel MALDI target plate using the sandwich method. When the CHCA matrix was used, 0.5 µl of the matrix was first applied on the plate and allowed to dry. A sample (0.5 µl) was applied and then immediately layered on top with the 0.5 µl of the matrix. In some cases, the sample was diluted fivefold in the CHCA matrix containing ammonium phosphate, and 1 µl of the sample and matrix mix was applied. The peptide mass spectrum was acquired using the Axima CFR plus the MALDI-TOF mass spectrometer in reflector mode. The instrument was set to pulsed extraction and the acceleration voltage is 20,000 V. Calibration standards used were bradykinin (757.39 Da), angiotensin II (1046.54 Da), P14R (1533.85 Da) and ACTH fragment 18–39 (2465.19 Da).

The monoisotopic peak list was generated using Kratos LAUNCHPAD software version 2.4 without using the smoothing function. Peak filters were applied to exclude masses lower than 750 Da. Mass peaks with the signal to noise ratio of 20 and above was used for the database search. MASCOT version 2.1 and 2.2 was used for database search. Database search parameters were as follows: tolerance 0.05–1 Da; species *Homo sapiens* and/or fungus; and maximum number of missed cleavages set to one for all samples. Fixed modifications used were carbamidomethyl C (for cysteine modification by iodoacetamide) and variable modifications used were oxidation of methionine and propionamide (for cysteine modification by acrylamide). Databases used for the search were NCBInr, MSDB, and Swissprot. The MASCOT score was considered significant if p<0.05.

### Statistical analysis

The significance of spot differences was calculated according to the student’s *t* test and the nonparametric two-tailed Mann–Whitney test with a confidence interval at 95%, and a p value of less than 0.05 was considered significant. The mean and median values of in vitro studies are based on three replicates.

## Results

**Figure 1:**
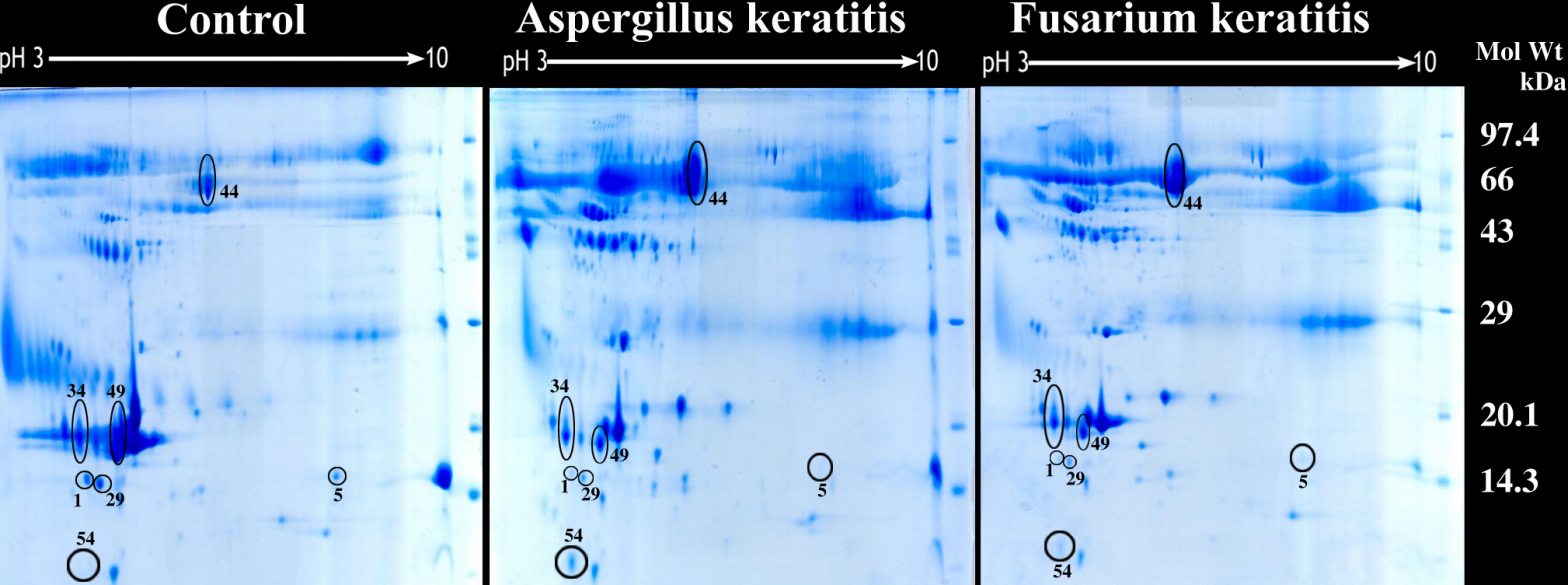
Representative 2DE gel map of tear proteins of normal subjects and fungal keratitis patients. Tear proteins (270 μg) were separated using an 18 cm pH 3–10 NL IPG strip in the first dimension and 12.5% SDS–PAGE followed by coomassie blue G-250 staining in the second dimension. The identified normal tear proteins, cystatin S precursor (spot 1), cystatin SN precursor (spot 5), cystatin (spot 29), human tear lipocalin (spot 49), prolactin inducible protein (spot 34), serum albumin (spot 44), and fungal protein such as the glutaredoxin-related protein (spot 54) are marked and compared between control tear samples, *Fusarium* keratitis tear samples, and *Aspergillus* keratitis tear samples.

Figure 1 shows the 2DE gel pattern of proteins from controls as well as from *Aspergillus*-infected and *Fusarium*-infected tear samples. The identified protein spots from silver stained gels were selected and compared using the ImageMaster 2D platinum version 6.0 software (GE healthcare) and quantified. A total of 63 spots could be detected in the two gels. Among the 63 spots, the difference could be measured for 46 spots. The presence of highly abundant proteins precludes the resolution of additional spots.

**Figure 2:**
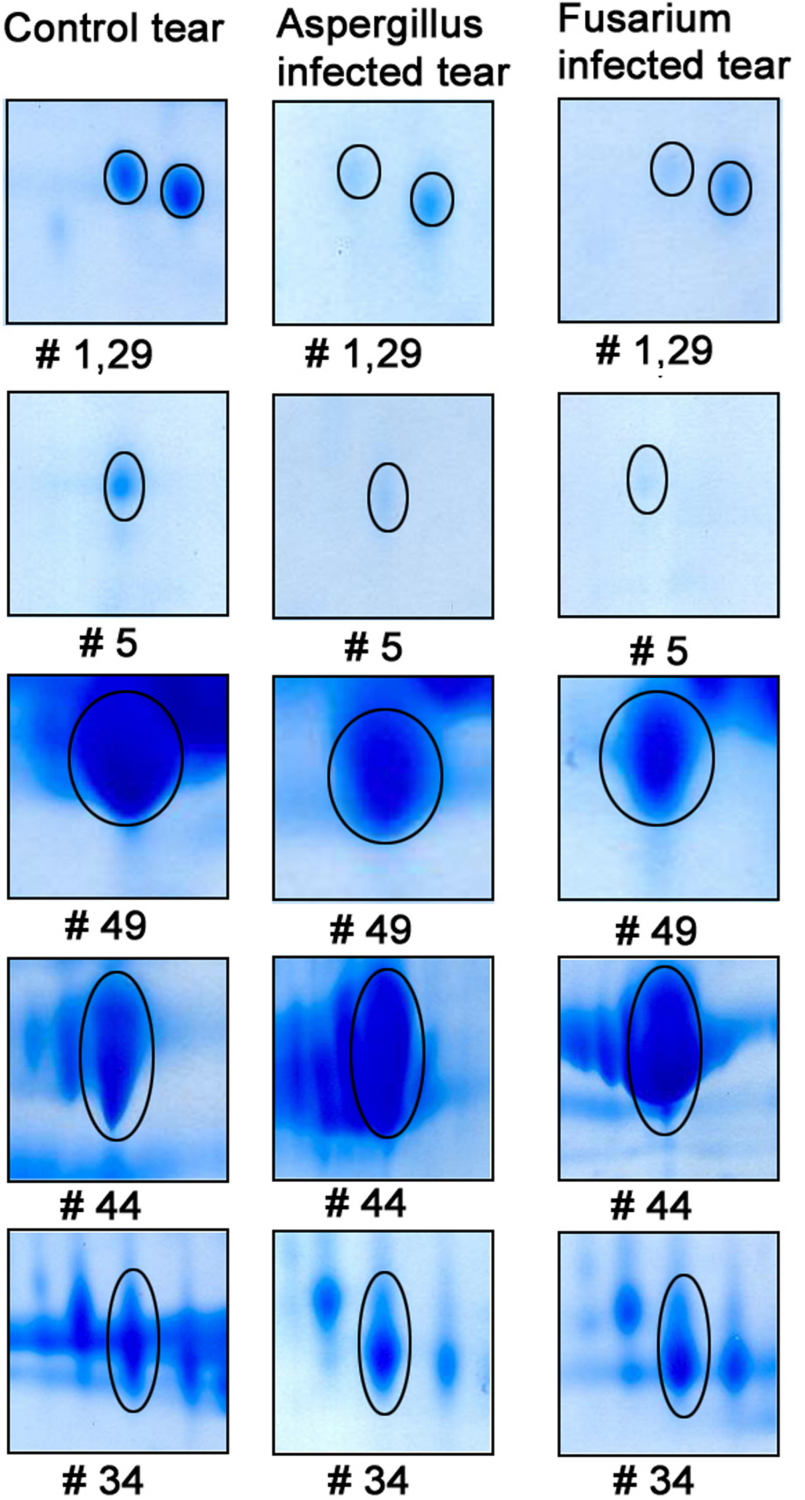
Magnified 2DE map of spots which were differentially expressed in the tears of mycotic keratitis patients. Among six normal tear proteins identified, four proteins (cystatin S precursor [1], cystatin SN precursor [5], cystatin [29], and human tear lipocalin [49]) were downregulated and two proteins (prolactin inducible protein [34] and serum albumin precursor [44]) were upregulated in fungal keratitis” in “Among six normal tear proteins identified, four proteins (cystatin S precursor, cystatin SN precursor, cystatin, and human tear lipocalin) were downregulated and two proteins (prolactin inducible protein and serum albumin precursor) were upregulated in fungal keratitis.

The glutaredoxin-related protein was found to be expressed only in the infected tear samples and was absent in the control tear samples. The expression of this protein was higher in the tear samples obtained from *Aspergillus* keratitis than in the tear samples obtained from *Fusarium* keratitis. In addition, six other normal tear proteins were also shown to be differentially regulated. Among the six normal tear proteins, prolactin inducible protein and serum albumin precursor were upregulated while cystatin S precursor, cystatin SN precursor, cystatin, and human tear lipocalin were downregulated in the infected tear when compared to the control tear sample (Figure 2).

### Differential expression of tear proteins

In analyzing differentially expressed spots in the control tear samples and the *Fusarium* tear and *Aspergillus* tear samples, 11 spots were upregulated in the *Fusarium* tear samples when compared to the control tear samples and eight spots were upregulated in the *Aspergillus* tear samples when compared to control tear samples. To minimize the error in calculating the intensity difference of spots between the gels, the upregulation was analyzed by calculating the ratio of percent intensity across all replicate gels. Among the 11 spots upregulated in the *Fusarium* tear samples, variation of expression level of seven were statistically significant through the Mann–Whitney test and four spots through the Student’s *t*-test distribution. Among the eight spots upregulated in the *Aspergillus* tear samples, upregulation of six spots were statistically significant through Mann–Whitney test and two spots through the Student’s *t-*test distribution.

## Discussion

Our findings show that tears can be used as a clinical source to study the proteomic responses in patients with fungal keratitis. The presence of glutaredoxin-related protein in the tears of patients with fungal keratitis implies that the fungus undergoes stress-related gene expression during infection [33]. The 2DE gel map showed significant differences in the protein expression between the control and infected tear samples. Further analysis will give insight into the pathogenesis of the disease.

### Tear protein profile by 2DE analysis

Like most techniques, the quality of the 2DE analysis depends on the preparation of the sample. Since the volume of the tear sample is usually limited to few microliters, the samples were pooled in some experiments and were precipitated to remove salts. Sufficient resolving power and reproducibility are also needed for comparative proteome analysis. Our electrophoretic procedure and staining method, which is described under the Methods section, were optimized for the analysis of tear proteome using 2DE. The removal of highly abundant proteins is essential to further improve the resolution. Even with the current gels, we could clearly show the variation in expression of selected spots with statistical confidence. Each sample was examined at least four times to ensure the variability of the observed changes.

### Significance of glutaredoxin-related protein

The glutaredoxin-related protein was found to be expressed only in the infected tear samples and absent in the control tear samples. Glutaredoxins (GRXs) are oxidoreductases that protect cells against oxidative stress [34,35] and are required for the activity of specific enzymes [36]. GRX has been found in bacteria, yeasts, and higher eukaryotes [36,37]. These are ubiquitous small heat-stable oxidoreductases that are involved in many cellular processes, including deoxyribonucleotide synthesis, the repair of oxidatively damaged proteins, protein folding, and sulfur metabolism [35]. GRXs contain cysteine residues in the conserved region [38,39] and can be subdivided into two subfamilies, dithiol GRXs with the carboxypeptidase Y/ferrichrome (CPY/FC) active site motif and monothiol GRXs with the CGFS motif. Both subfamilies share a thioredoxin-fold structure. Some monothiol GRXs exist with a single-Grx domain while others have a thioredoxin-like domain (Trx) and one or more Grx domain in tandem. Most fungi have both dithiol and monothiol GRXs with different subcellular locations. GRX-like molecules also exist in fungi that differ by one residue from one of the canonical active site motifs.

Glutaredoxin is also known to be expressed by *Aspergillus* under oxidative stress conditions [33]. The major antioxidant molecules and interconnecting pathways are active in *Aspergillus*, and it has the entire armamentarium to combat oxidative stress [33,40]. Thus, the presence of this glutaredoxin-related protein only in the fungal-infected tear samples indicates that it could be a fungal protein. A Mascot search against the *Homo sapiens* database did not give any hit for the MALDI data whereas the fungal database gave an excellent Mascot score. Interestingly, the protein expression was higher in the *Aspergillus*-infected tear sample than in the *Fusarium*-infected tear sample. Further studies are needed with a large number of samples to give insight about the contribution of this protein to the pathophysiological process of mycotic keratitis. Further analysis using tear samples collected from various stages of fungal keratitis is in progress to understand the validation of using this protein as a marker associated with the severity, progression, or therapeutic response of keratitis.

### Functional implication of differentially expressed proteins

The up and downregulation of proteins can provide important information about disease processes and can lead to a better understanding of the pathogenesis. Furthermore, this process might identify important biomarkers of the disease and thus will lead to predictive medicine.

Table 1 describes the functional activity of identified proteins. Since the levels of some identified normal tear proteins decreased in the patient group, the downregulation of these proteins may affect the function of tear film. The first line of protection of the eye depends on the nature of the protein components present in the secretion. Besides secretory IgA, four proteins (lysozyme, lactoferrin, cystatin, and tear lipocalin) make up nearly 50% of the protein.

**Table 1:** Functional activity of identified proteins and its regulation in the fungal keratitis patient.

**Tear proteins**	**Spot number in 2D gel**	**Mowse score/** **significant score**	**Regulation in infected tear**	**Function**
Cystatin S precursor	1	122 (>64)	Downregulation	Cysteine protease inhibitor
Cystatin SN precursor	5	66 (>55)	Downregulation	Cysteine protease inhibitor
Cystatin	29	67 (>60)	Downregulation	Cysteine protease inhibitor
Human tear lipocalin	49	72 (>64)	Downregulation	Lipid scavenging and transport to outer tear layer
Prolactin inducible protein	34	92 (>64)	Upregulation	Suppressing T-cell apoptosis
Serum albumin precursor	44	112 (>67)	Upregulation	Regulation of the colloidal osmotic pressure of blood
Glutaredoxin-related protein	54	58 (>65)	Upregulation	Protect cells against oxidative stress

Six normal tear proteins were identified in healthy subjects and fungal keratitis patients with varied regulation. Glutaredoxin related protein was expressed only in fungal keratitis patients. The identified proteins are listed in the order of their regulations.

The role of cystatins as proteinase inhibitors is well established. The balance between protease and protease inhibitor is believed to play a significant role in maintaining the normal ocular surface condition [41].Cystatins are generally tight-binding inhibitors of cysteine proteases and have a protective function by regulating the activities of endogenous cysteine proteases causing uncontrolled proteolysis and tissue damages [42-45]. The reduction of cystatins in the tears of patients with fungal keratitis correlates well with their functional role since cysteine protease inhibitors were demonstrated to be reduced in several pathological conditions [46].

The tear lipocalin protein level in human tears ranges between 0.5 and 1.5 g/l, making up 10%–20% of the total tear protein content in tears and is considered tear-specific [47-49].The origin and concentration of lipocalin in tears may reflect an important function of this protein on the ocular surface [50]. The possible role for lipocalin is the transport of lipids [51]. It is previously called tear-specific prealbumin [52]. It has been shown that lipocalin is synthesized in the main and accessory lacrimal glands. Studies on tear samples from normal subjects and keratoconjunctivitis sicca patients showed that lipocalin may serve as the marker protein for lacrimal gland functions.

Prolactin inducible protein (PIP) has been found in tears, saliva, sweat, seminal plasma, submucosal gland of the lung, and amniotic fluid and was shown to be involved in local immune function. It is also capable of suppressing T-cell apoptosis in the reproductive system via high-affinity interaction with CD4 [23].

Overall, in this study, the 2DE profile of fungal keratitis patients were compared to those of healthy volunteers and downregulated proteins were found which are associated with the disease. The presence of the glutaredoxin-related protein exclusively in the tears of patients with fungal keratitis indicates that the fungus might undergo stress-related gene expression during infection. The tear proteomic analysis could be applied to understand the disease mechanism and to find new diagnostic or therapeutic targets as well as classification of disease state.
